# Adverse event reporting of the IGF-1R monoclonal antibody teprotumumab: a real-world study based on the US food and drug administration adverse event reporting system

**DOI:** 10.3389/fphar.2024.1393940

**Published:** 2024-08-09

**Authors:** Jiawei Zhao, Yong Tao

**Affiliations:** Department of Ophthalmology, Beijing Chaoyang Hospital, Capital Medical University, Beijing, China

**Keywords:** IGF-1R monoclonal antibody, thyroid eye disease, FAERS, teprotumumab, adverse events

## Abstract

**Background:**

Teprotumumab, an IGF-1R monoclonal antibody, has shown significant efficacy in treating thyroid eye disease (TED). However, since teprotumumab was launched in 2020 and first approved in the United States, there were limited reports of post-marketing adverse events (AEs). In this study, we aimed to mine and analyze the AEs signals with teprotumumab on the basis of the United States Food and Drug Administration (FDA) Adverse Event Reporting System (FAERS) to provide instructions in clinical practice concerning adverse reactions and assistance in drug development and import/export into other countries.

**Methods:**

All AE reports were obtained from the FAERS database from the first quarter of 2020 to the fourth quarter of 2023. To comprehensively analyze the AEs, we applied four disproportionality analysis algorithms, including the reporting odds ratio (ROR), the proportional reporting ratio (PRR), the Bayesian confidence propagation neural network (BCPNN), and the multi-item gamma Poisson shrinker (MGPS) algorithms.

**Results:**

A total of 687 reports from 200 patients related to administration of teprotumumab were obtained, and 78% of the cases was female. Signal detection of teprotumumab at the system organ class (SOC) level included gastrointestinal disorders, ear and labyrinth disorders, general disorders and administration site conditions, nervous system disorders, and musculoskeletal and connective tissue disorders. AEs that ranked top five at the preferred terms (PTs) level were muscle spasms, fatigue, tinnitus, headache, and deafness. The median time to those AEs onsets was 48 days (interquartile range 19.0–92.0 days) after administering drugs. Additionally, our results indicated the AEs in reproductive system and breast disorders because the prevalence of TED was more common in women.

**Conclusion:**

This study identified many AEs associated with teprotumumab and unveiled potential new AE signals. These results can provide valuable evidence for further clinical application of teprotumumab and are important in enhancing clinical medication safety.

## Introduction

Thyroid eye disease (TED) is an autoimmune inflammatory disorder that affects the orbit and extraocular muscles, which is commonly associated with Graves’ disease. TED is most frequently associated with hyperthyroidism, especially Graves’ disease, which accounts for about 90% of the cases. However, about 10% of patients with TED have either a normal-functioning or under-functioning thyroid (Hoang et al., 2022). The mean annual incidence rate of TED was 16 cases per 100,000 people in women, and 3 cases per 100,000 people in men, resulting in a 4:1 ratio of women to men with TED (Smith et al., 2016; Shen et al., 2023). Environmental factors, which includes infection, smoking, stress, air pollution, and some genetic factors may play a role in the susceptibility, development, and worsening of TED (Cao et al., 2023; Grixti et al., 2023; Mohamed et al., 2023). The pathogenesis of TED involves the loss of immune tolerance to thyroid-stimulating hormone receptor (TSHR) and insulin-like growth factor-1 receptor (IGF-1R), along with activation of T cells and B cells. The overexpression of IGF-1R appears central to disease pathogenesis (Patel et al., 2019). In addition, the infiltration and activation of orbital fibroblasts could secrete pro-inflammatory cytokines and glycosoaminoglycans, leading to orbital inflammation, adipogenesis, muscle enlargement, retro-orbital expansion with resultant exophthalmosis particularly pathognomonic for TED (Khong et al., 2016; Zheng et al., 2022; Cui X. et al., 2023).

Currently used therapeutic options for TED include medical treatments, such as glucocorticoids, immunosuppressants, and radioactive iodine, and these treatments are not particularly effective on the progressive outcomes of proptosis and diplopia. However, these drugs may have various side effects, such as osteoporosis, diabetes, hypertension, infection, mood disorders, liver toxicity, bone marrow suppression, allergic reactions, and radiation-induced malignancies (Hoang et al., 2022; Swaify et al., 2023). Other therapeutic options include surgical interventions, such as orbital decompression, strabismus surgery and eyelid surgery. Unavoidably, surgical interventions normally come with complication, including infection, scarring, nerve damage, vision loss, and recurrence of proptosis or diplopia (Baeg et al., 2023). Teprotumumab is a monoclonal antibody that inhibits the IGF-1R, which is closely associated with the pathogenesis of TED. It is the first and only Food and drug administration (FDA)-approved drug for the treatment of TED based on two clinical trials (Smith et al., 2017; Douglas et al., 2020). The pooled analysis of these clinical trials showed that integrated responses for composite outcome of proptosis and diplopia were observed after final dose at 7 weeks and 51 weeks in 92% and 83% of patients, respectively (Kahaly et al., 2021). Another randomized double-masked, placebo-controlled trial conducted at 11 US centers showed 61.9% of patients in the teprotumumab group had a proptosis response and significantly greater improvements from baseline in the Graves’ Ophthalmopathy Quality of Life Visual Function subscale compared to placebo. While for enrolled patients with diplopia at baseline, proportions were unevenly matched in the teprotumumab (33%) and placebo arms (20%) (Kahaly et al., 2021; Douglas et al., 2024).

Although teprotumumab is currently recognized as the preferred medication for the treatment of TED, repeated injections of teprotumumab still can inevitably cause some complications. The most common side effects of teprotumumab are muscle spasms, nausea, alopecia, diarrhoea, fatigue, hyperglycaemia, hearing impairment, dysgeusia, headache, dry skin, and rash (Kahaly et al., 2021). Adverse events spontaneous reporting system is one of the most currently important methods in monitoring the safety of medicinal products. FDA Adverse Event Reporting System (FAERS) is a public database designed to support the FDA’s post-marketing safety surveillance program for drug and therapeutic biologic products through a system of spontaneous reports by consumers, health professionals, drug manufacturers, and other non-healthcare workers. Recently published paper reported the top ten PTs ranked by the frequency of reporting and concluded that clinical application of teprotumumab should be closely monitored for ototoxicity, nail abnormalities, and menstrual changes using the reporting odds ratio (ROR) (Wang X.-L. et al., 2024). In this study, we made a comprehensive analysis of the AEs of teprotumumab in FAERS dataset using four algorithms, including ROR, and proportional reporting ratio (PRR). Bayesian Statistics on the other hand, included bayesian confidence propagation neural network (BCPNN) and multi-item gamma Poisson shrinker (MGPS) for better sensitivity and specificity in detecting signals. In this study, we focused on breaking out the reporting of events of special interest, and compared the data from two clinical trials with our results. Based on the needs of clinical, rational and precise drug use and protection of patients’ rights and interests, we evaluated each AE reports of teprotumumab using FAERS database. Findings of this study create real-world evidence for risk signal detection and guide future comparative effectiveness and post-marketing surveillance research for teprotumumab.

## Methods

### Data source

**FIGURE 1:**
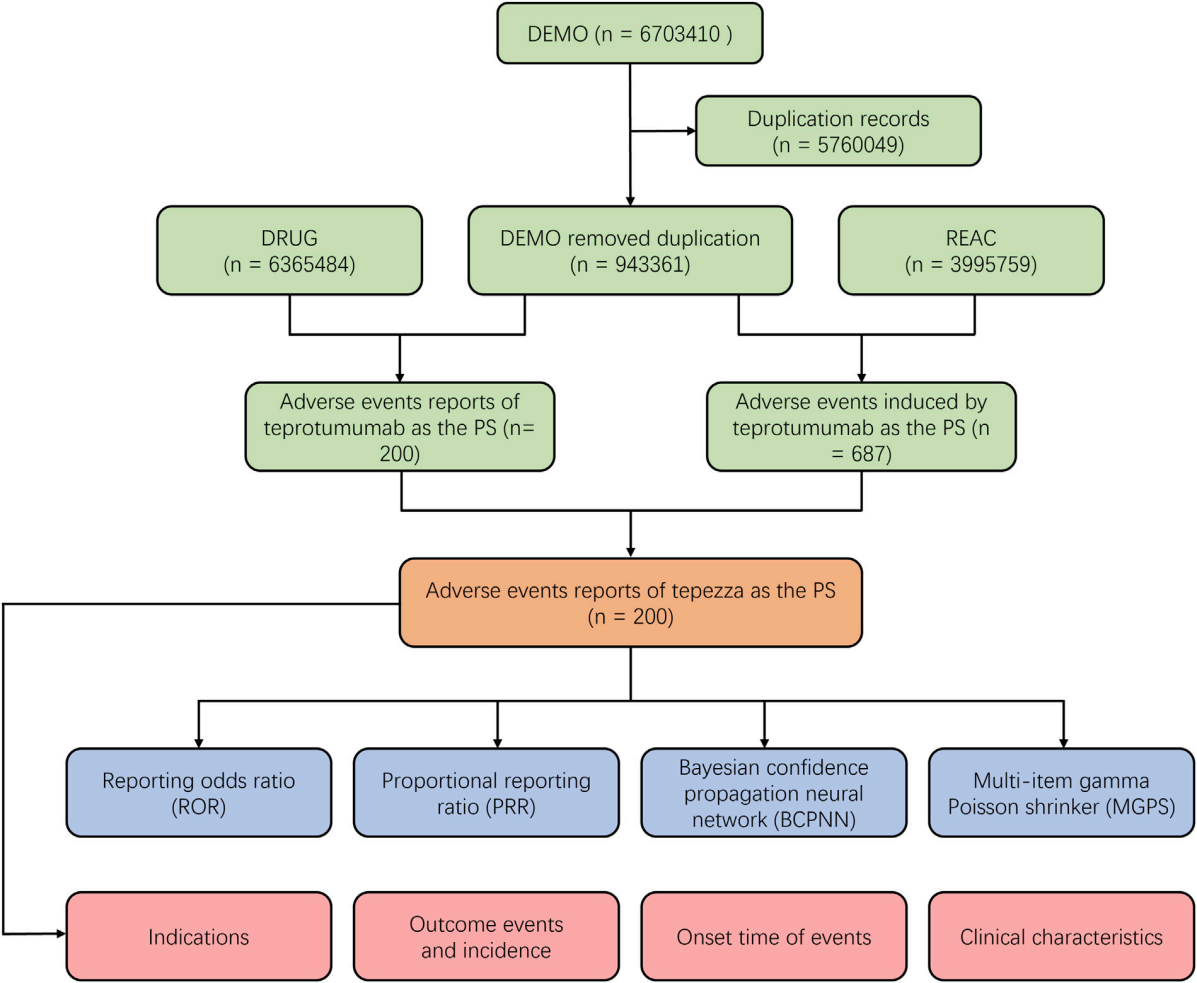
Scheme of the workflow of selection of teprotumumab-related adverse events from the FAERS database.

To systematically evaluate the safety of teprotumumab in the post-marketing period, we conducted a retrospective pharmacovigilance study using data obtained from the FAERS database. The FAERS database covers data from the first quarter of 2020 to the fourth quarter of 2023 and can be accessed at (https://fis.fda.gov/extensions/FPD-QDE-FAERS/FPD-QDE-FAERS.html). The FAERS database uses the PTs from the Medical Dictionary for Regulatory Activities (MedDRA) to classify AEs. These PTs are organized into broader categories such as System Organ Class SOC. These categories group terms based on aspects like anatomy, pathology, physiology, etiology, or function, providing a structured hierarchy to define specific organs or systems affected by adverse events. To eliminate duplicate reports, as recommended by the FDA, we sorted the DEMO table’s PRIMARYID, CASEID, and FDA_DT. We retained the report with the largest FDA_DT for identical CASEID, and in cases of identical CASEID and FDA_DT, the report with the largest PRIMARYID was kept (Figure 1).

### Drug identification and adverse events

As FAERS had two variables including DRUGNAME and PROD_AI, both the brand names and common names were employed to recognize records related to teprotumumab. In this study, “TEPROTUMUMAB TRBW”, “TEPEZZA”, “TEPEZZA INJ”, “TEPEZZA 2000 MG IV IN 250 ML 0 9 NACL”, “RO4858696 INVESTIGATIONAL IGF 1R ANTAGONIST”, “RO4858696 PLACEBO”, “RO4858696”, “RO4858696 INJECTION FOR INFUSION”, “RO4858696 IGF 1R ANTAGONIST”, “RO4858696 R1507”, “R1507 R04858696” were used to search. To enhance accuracy, the role code of AEs was retained only as the primary suspect (PS) drug (Cui Z. et al., 2023; Zhang et al., 2023). Furthermore, the time-to-onset (TTO) of AEs caused by teprotumumab were defined as the interval between EVENT_DT (AEs onset date, in DEMO file) and START_DT (start date of teprotumumab use, in THER file) (Jiang et al., 2023).

### Data mining algorithm

One of the most frequently used methods of safety signal detection is disproportionality analysis, which consisted of two categories: Frequentist Statistics and Bayesian Statistics. Frequentist Statistics included ROR, and proportional reporting ratio (PRR). Bayesian Statistics on the other hand, included bayesian confidence propagation neural network (BCPNN) and multi-item gamma Poisson shrinker (MGPS). The corresponding ROR, information components (IC), and 95% confidence interval (CI) were calculated accordingly to determine the signal intensity of each adverse event for each drug (Hauben and Bate, 2009; Suling and Pigeot, 2012; Liu et al., 2013; Park et al., 2020). The frequentist method had its characteristics: the sensitivity of frequency method was high, but it was easy to produce false positive signals when the number of reports was small. The ratio imbalance measurement algorithm was shown in Table 1. The principle of disproportionate measure and standard of signal detection were shown in Table 2.

**TABLE 1:** Ratio imbalance measurement algorithm.

	AEs of interest	All other AEs	Total
Drug of interest	a	b	a+b
All other drugs	c	d	c + d
Total	a+c	b + d	a+b + c + d

a, Number of reports that contain both targeted drug and targeted drug adverse reactions; b, Number of reports of other drug adverse reactions that contain the targeted drug; c, Number of reports of targeted drug adverse reactions that contain other drugs; d, Number of reports that contain other drugs and other drug adverse reactions; All other drugs, all drugs in the FAERS, dataset other than teprotumumab.

**TABLE 2:** Summary of major algorithms used for signal detection.

Algorithms	Equation
ROR	ROR = ad/b/c
	95%CI = e^ln(ROR)±1.96(l/a+1/b+l/c+l/d) ^0.5^
PRR	PRR = a(c + d)/c/(a+b)
	x^2^ = [(ad-bc) ^2](a+b + c + d)/[(a+b) (c + d) (a+c) (b + d)
BCPNN	IC = logza(a+b + c + d)/((a+c) (a+b))
	95%Cl = E(IC)±2V(IC) ^0.5
MGPS	EBGM = a(a+b + c + d)/(a+c)/(a+b)
	95%CI = e^ln(EBGM)±1.96(l/a+l/b+l/c+l/d) ^0.5^

ROR, reporting odds ratio; PRR, proportional reporting ratio; BCPNN, bayesian confidence propagation neural network; MGPS, Multi-item gamma Poisson shrinker; 95% CI, 95% confidence interval; EBGM, empirical Bayesian geometric mean.

### Statistical analysis

Four algorithms including ROR, PRR, BCPNN, and MGPS were used to indicate the association between the AEs and target drug. ROR is a disproportionality measure that employs logistic regression to estimate the likelihood of reporting an adverse event associated with a specific drug relative to all other drugs. It takes into consideration the total number of reports and has the capability to adjust for potential confounding variables. Like ROR, the PRR is a straightforward metric employed to identify potential signals of adverse drug reactions. It assesses whether the proportion of a specific adverse event reported with a particular drug exceeds the proportion of the same event reported across all other drugs. BCPNN is an advanced algorithm employing Bayesian principles to assess the likelihood of a causal connection between a drug and an adverse event. It is adept at managing sparse data and is generally less prone to generating false signals compared to PRR. The algorithm operates by propagating evidence strength across a network of nodes that represent drugs and events, continuously updating the probability of an association based on accumulated evidence from interconnected nodes. MGPS is a shrinkage method utilized to modify effect sizes, akin to PRR or ROR, with the aim of minimizing false-positive signals. It applies a gamma distribution to observed counts and subsequently adjusts estimates towards a central value, typically zero. This approach aids in emphasizing the most probable signals for further investigation. Each of these algorithms possesses unique strengths and limitations, and their selection depends on the requirement to strike a balance between sensitivity and specificity in detecting signals (Shu et al., 2022; Wang K. et al., 2024).

## Results

### General characteristics in the real-world population

**FIGURE 2:**
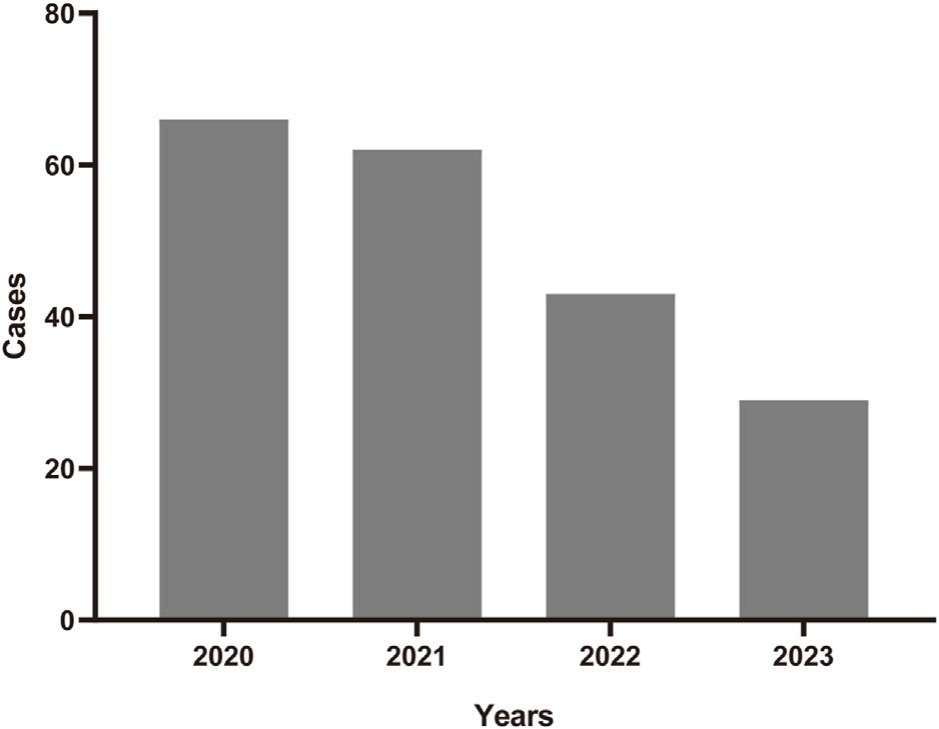
The annual distribution of teprotumumab-related AE reports from 2020 to 2023.

A total number of 6,703,410 AEs related to administration of teprotumumab were documented in the FAERS database dated from Q1 2020 to Q4 2023 (Figure 1). It is notable that there were more female patients reported (78.00%) compared to male patients (22.00%) due to higher woman prevalence over man, which is consistent with the fact that Graves’ Disease is female predominate (Table 3). The reported proportions of body weight in the categories of <50 kg, 50–100 kg, and >100 kg was 4.50%, 81.00%, and 14.50%, respectively. A higher occurrence of teprotumumab-related AEs was observed in the category of 18–64.9 years old (63.00%) compared to elderly patients (65–85 years old, 25.50%). Almost all the AEs reports were from the United States (98.50%), mainly because, as a newly developed and marketed drug, teprotumumab was first approved in the United States. From an overall perspective, the number of AEs reports has been decreasing between the year of 2020 and 2023 (Figure 2).

**TABLE 3:** Clinical characteristics of reports with teprotumumab from the FAERS Database (January 2020–December 2023).

Characteristics	Case number	Proportion (%)
**Number of events**	200	
Gender		
Female	156	78.00
Male	44	22.00
Weight (kg)		
<50	9	4.50
>100	29	14.50
50∼100	162	81.00
Age (years)		
<18	2	1.00
>85	1	0.50
18∼64.9	126	63.00
65∼85	51	25.50
Missing	20	10.00
Reporter country		
United States	197	98.50
Country not specified	3	1.50

### Signal detection of teprotumumab at the system organ class level

**FIGURE 3:**
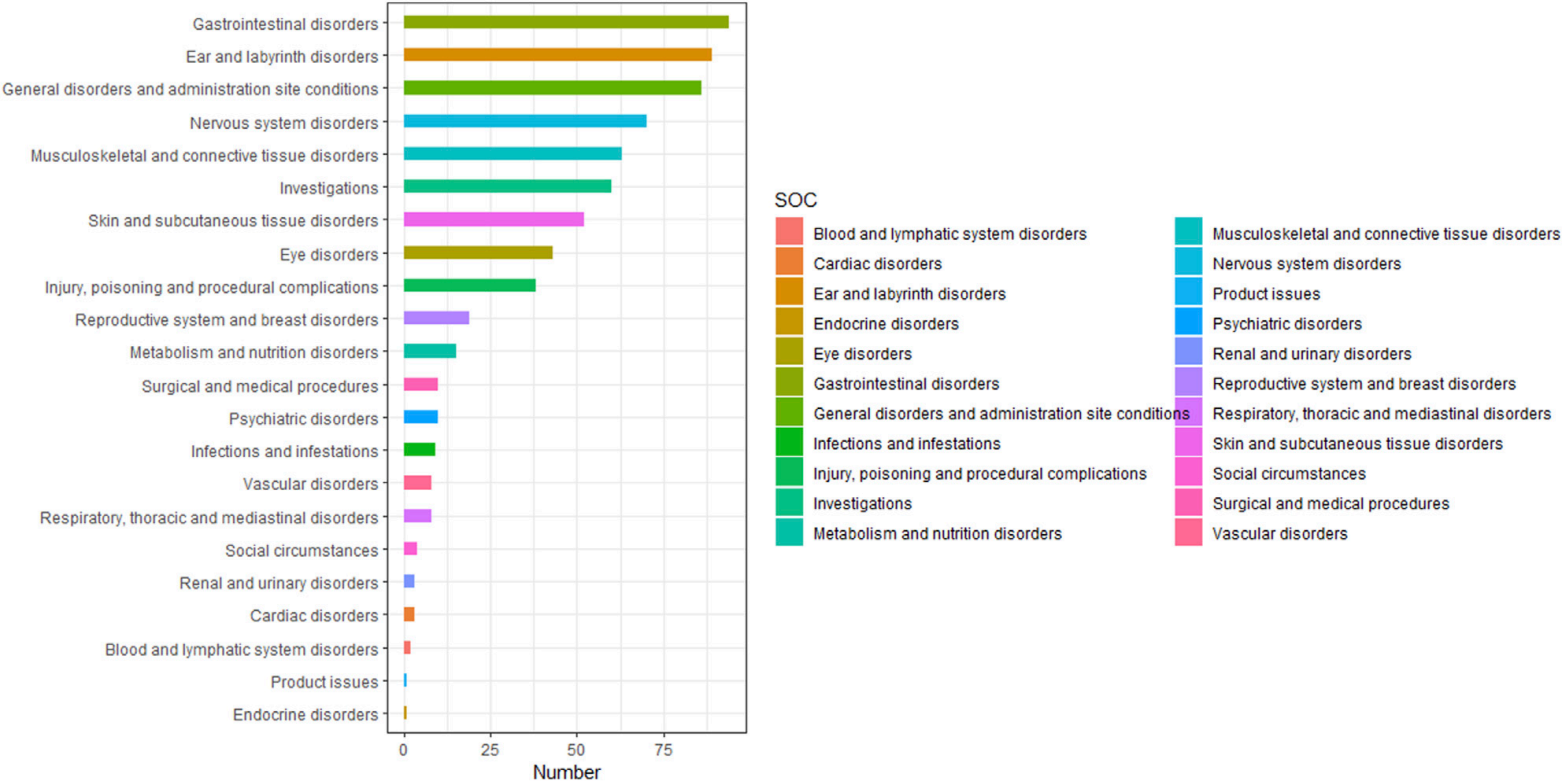
Bar plot showing the frequency of occurrences categorized by SOC.

**FIGURE 4:**
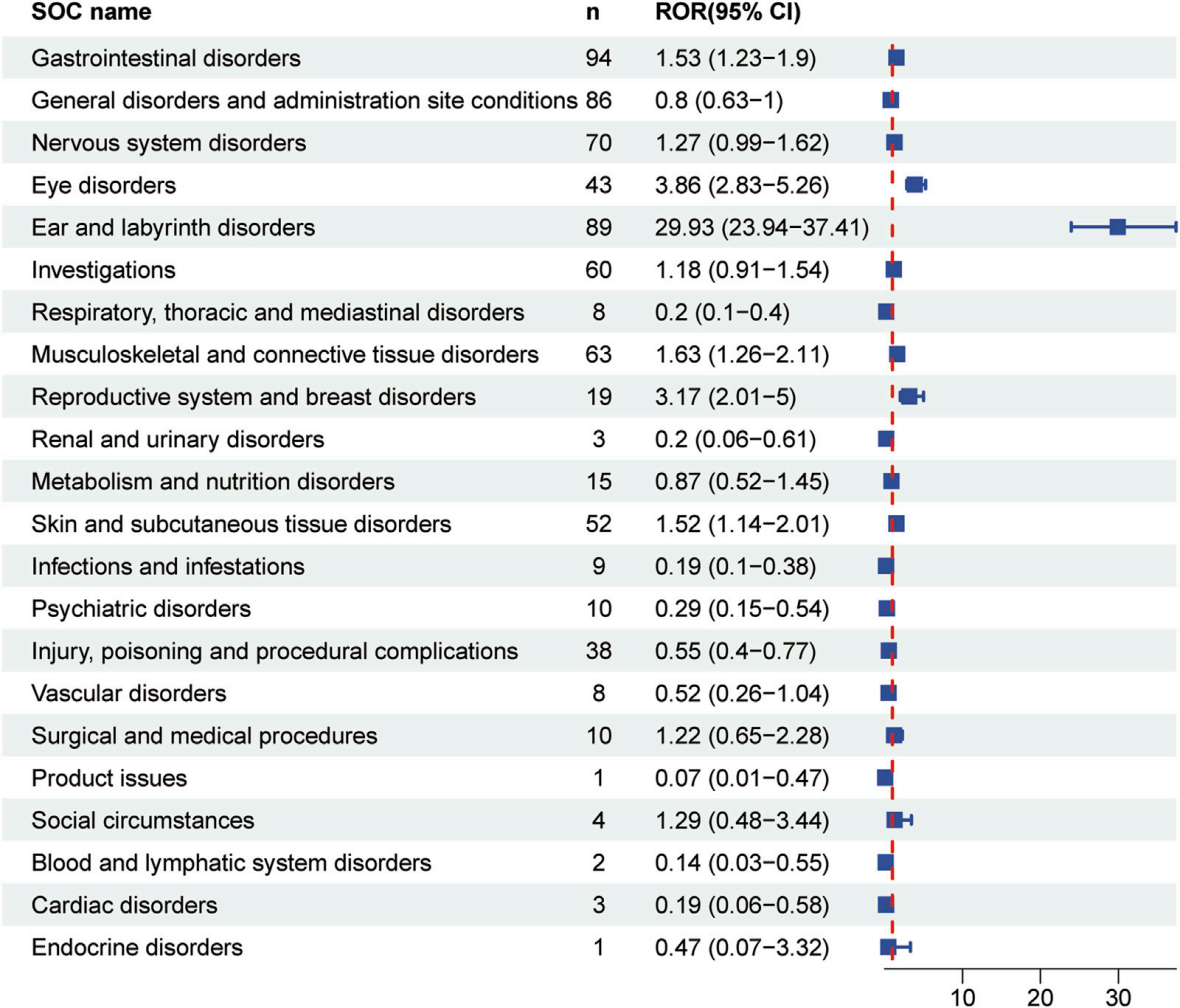
Forest plot showing reporting odds ratios (RORs) for teprotumumab-related AEs at the SOC levels. 95% CI indicates 95% confidence interval.

Table 4 presented the signal strength and number of reports for teprotumumab at the System Organ Class (SOC) level. Our statistical analysis identified a total of 22 organ systems were implicated in teprotumumab-induced AEs. The top 5 SOC with teprotumumab-related AEs was gastrointestinal disorders (SOC code: 10017947, 13.66%), ear and labyrinth disorders (SOC code: 10013993, 12.94%), general disorders and administration site conditions (SOC code: 10018065, 12.50%), nervous system disorders (SOC code: 10029205, 10.17%), and musculoskeletal and connective tissue disorders (SOC code: 10028395, 9.16%), accounting in total for 58.43% of all the reports (Figures 3, 4). Notably, ear and labyrinth disorders ranked first among all the SOC categories, with a ROR to be 29.93 (23.94–37.41), indicating a strong relationship with teprotumumab. Also, it was worth noticing that some other significant SOCs existed including eye disorders (SOC code: 10015919, 6.25%) considering that teprotumumab was used to treat eye diseases. In addition, we focused on the reproductive system and breast disorders (SOC code: 10038604, 2.76%) because clinically TED was more common in women.

**TABLE 4:** Signal strength of AEs of teprotumumab at the SOC level in FAERS database. ROR, reporting odds ratio. PRR, proportional reporting ratio. IC, information component. EBGM, empirical Bayesian geometric mean.

SOC name	n	%	ROR (95%Cl)	PRR (χ2)	IC (IC025)	EBGM (EBGM05)
Gastrointestinal disorders	94	13.66	1.53 (1.23–1.9)	1.45 (14.67)	0.54 (−1.13)	1.45 (1.17)
Ear and labyrinth disorders	89	12.94	29.93 (23.94–37.41)	26.19 (2157.08)	4.7 (3.03)	26.07 (20.86)
General disorders and administration site conditions	86	12.50	0.8 (0.63–1)	0.82 (3.95)	−0.28 (−1.95)	0.82 (0.66)
Nervous system disorders	70	10.17	1.27 (0.99–1.62)	1.24 (3.55)	0.31 (−1.36)	1.24 (0.97)
Musculoskeletal and connective tissue disorders	63	9.16	1.63 (1.26–2.11)	1.57 (14)	0.65 (−1.02)	1.57 (1.21)
Investigations	60	8.72	1.18 (0.91–1.54)	1.17 (1.58)	0.22 (−1.44)	1.17 (0.9)
Skin and subcutaneous tissue disorders	52	7.56	1.52 (1.14–2.01)	1.48 (8.48)	0.56 (−1.11)	1.48 (1.11)
Eye disorders	43	6.25	3.86 (2.83–5.26)	3.68 (85.37)	1.88 (0.21)	3.68 (2.7)
Injury, poisoning and procedural complications	38	5.52	0.55 (0.4–0.77)	0.58 (12.89)	−0.79 (−2.46)	0.58 (0.42)
Reproductive system and breast disorders	19	2.76	3.17 (2.01–5)	3.11 (27.41)	1.64 (−0.03)	3.11 (1.97)
Metabolism and nutrition disorders	15	2.18	0.87 (0.52–1.45)	0.87 (0.29)	−0.2 (−1.87)	0.87 (0.52)
Psychiatric disorders	10	1.45	0.29 (0.15–0.54)	0.3 (17.35)	−1.74 (−3.41)	0.3 (0.16)
Surgical and medical procedures	10	1.45	1.22 (0.65–2.28)	1.22 (0.39)	0.28 (−1.39)	1.22 (0.65)
Infections and infestations	9	1.31	0.19 (0.1–0.38)	0.21 (29.55)	−2.28 (−3.95)	0.21 (0.11)
Respiratory, thoracic, and mediastinal disorders	8	1.16	0.2 (0.1–0.4)	0.21 (25.17)	−2.25 (−3.92)	0.21 (0.1)
Vascular disorders	8	1.16	0.52 (0.26–1.04)	0.53 (3.5)	−0.93 (−2.6)	0.53 (0.26)
Social circumstances	4	0.58	1.29 (0.48–3.44)	1.28 (0.25)	0.36 (−1.31)	1.28 (0.48)
Renal and urinary disorders	3	0.44	0.2 (0.06–0.61)	0.2 (9.77)	−2.32 (−3.99)	0.2 (0.06)
Cardiac disorders	3	0.44	0.19 (0.06–0.58)	0.19 (10.72)	−2.41 (−4.08)	0.19 (0.06)
Blood and lymphatic system disorders	2	0.29	0.14 (0.03–0.55)	0.14 (10.88)	−2.85 (−4.52)	0.14 (0.03)
Product issues	1	0.15	0.07 (0.01–0.47)	0.07 (13.09)	−3.88 (−5.55)	0.07 (0.01)
Endocrine disorders	1	0.15	0.47 (0.07–3.32)	0.47 (0.61)	−1.1 (−2.77)	0.47 (0.07)

### Signal detection of teprotumumab at the preferred terms level

A total of 238 types of PTs was reported in different organ systems. We then ranked AEs according to the frequency of occurrence, and listed the AEs with a least frequency over 0.5% (Table 5). Top AEs to teprotumumab included muscle spasms (PT code: 10028334, 6.4%), fatigue (PT code: 10016256, 4.66%), tinnitus (PT code: 10043882, 3.78%), headache (PT code: 10019211, 3.78%), deafness (PT code: 10011878, 3.78%), nausea (PT code: 10028813, 2.91%), blood glucose increased (PT code: 10005557, 2.62%), infusion related reaction (PT code: 10051792, 2.62%), diarrhea (PT code: 10012735, 2.47%), alopecia (PT code: 10001760, 1.89%), dizziness (PT code: 10013573, 1.46%), condition aggravated (PT code: 10010264, 1.46%), blood pressure increased (PT code: 10005750, 1.46%), weight decreased (PT code: 10047895, 1.46%), ear discomfort (PT code: 10052137, 1.31%), hypoacusis (PT code: 10048865, 1.31%), amenorrhoea (PT code: 10001928, 1.02%), and dry skin (PT code: 10013786, 1.02%), which accounted for over 1% in the sum of all AEs.

**TABLE 5:** Signal strength of AEs of teprotumumab at the PTs level in FAERS database. ROR, reporting odds ratio. PRR, proportional reporting ratio. IC, information component. EBGM, empirical Bayesian geometric mean.

PTs	n	%	ROR (95%Cl)	PRR (χ2)	IC (IC025)	EBGM (EBGM05)
Muscle spasms	44	6.40	21.75 (16.02–29.53)	20.42 (812.42)	4.35 (2.68)	20.35 (15.76)
Fatigue	32	4.66	3.59 (2.52–5.12)	3.47 (57.03)	1.79 (0.13)	3.47 (2.58)
Tinnitus	26	3.78	39.92 (26.94–59.14)	38.45 (942.96)	5.26 (3.59)	38.2 (27.49)
Headache	26	3.78	3.59 (2.42–5.31)	3.49 (46.62)	1.8 (0.13)	3.49 (2.51)
Deafness	26	3.78	106.77 (71.92–158.52)	102.78 (2575.8)	6.66 (4.99)	101.01 (72.57)
Nausea	20	2.91	2.25 (1.44–3.51)	2.21 (13.45)	1.14 (−0.52)	2.21 (1.52)
Blood glucose increased	18	2.62	17.41 (10.89–27.82)	16.98 (270.26)	4.08 (2.41)	16.93 (11.44)
Infusion related reaction	18	2.62	9.74 (6.09–15.55)	9.51 (137.18)	3.25 (1.58)	9.49 (6.41)
Diarrhea	17	2.47	2.03 (1.26–3.29)	2.01 (8.7)	1.01 (−0.66)	2.01 (1.34)
Alopecia	13	1.89	6.08 (3.51–10.52)	5.98 (54.05)	2.58 (0.91)	5.98 (3.77)
Dizziness	10	1.46	1.7 (0.91–3.17)	1.69 (2.82)	0.75 (−0.91)	1.69 (1)
Condition aggravated	10	1.46	2.53 (1.35–4.72)	2.5 (9.08)	1.32 (−0.35)	2.5 (1.48)
Blood pressure increased	10	1.46	4.59 (2.46–8.57)	4.53 (27.62)	2.18 (0.51)	4.53 (2.69)
Weight decreased	10	1.46	2.18 (1.17–4.08)	2.17 (6.33)	1.12 (−0.55)	2.17 (1.28)
Ear discomfort	9	1.31	59.69 (30.82–115.59)	58.92 (507.39)	5.87 (4.19)	58.34 (33.56)
Hypoacusis	9	1.31	17.73 (9.18–34.26)	17.51 (139.82)	4.13 (2.46)	17.46 (10.06)
Amenorrhoea	7	1.02	54.24 (25.67–114.59)	53.7 (358.75)	5.73 (4.06)	53.21 (28.46)
Dry skin	7	1.02	8.03 (3.81–16.91)	7.96 (42.58)	2.99 (1.32)	7.95 (4.26)
Vision blurred	6	0.87	4.49 (2.01–10.03)	4.46 (16.13)	2.16 (0.49)	4.46 (2.27)
Diplopia	6	0.87	20 (8.94–44.73)	19.83 (106.96)	4.3 (2.63)	19.77 (10.08)
Glycosylated hemoglobin increased	6	0.87	30.13 (13.46–67.45)	29.88 (166.68)	4.89 (3.22)	29.73 (15.15)
Pain	6	0.87	0.87 (0.39–1.95)	0.88 (0.11)	−0.19 (−1.86)	0.88 (0.45)
Asthenia	6	0.87	1.24 (0.56–2.77)	1.24 (0.28)	0.31 (−1.36)	1.24 (0.63)
Hyperglycaemia	6	0.87	11.97 (5.36–26.77)	11.88 (59.7)	3.57 (1.9)	11.86 (6.05)
Gingival recession	6	0.87	258.43 (113.7–587.38)	256.18 (1460.73)	7.94 (6.25)	245.4 (123.45)
Taste disorder	6	0.87	12.31 (5.5–27.51)	12.21 (61.65)	3.61 (1.94)	12.18 (6.22)
Onychoclasis	6	0.87	75.25 (33.52–168.96)	74.61 (430.24)	6.2 (4.53)	73.67 (37.45)
Rash	6	0.87	1.25 (0.56–2.79)	1.25 (0.29)	0.32 (−1.35)	1.25 (0.64)
Therapy cessation	6	0.87	10.56 (4.72–23.6)	10.48 (51.37)	3.39 (1.72)	10.46 (5.34)
Abdominal discomfort	5	0.73	2.47 (1.02–5.95)	2.46 (4.33)	1.3 (−0.37)	2.46 (1.18)
Dysgeusia	5	0.73	5.83 (2.42–14.06)	5.8 (19.85)	2.53 (0.86)	5.79 (2.77)
Weight increased	5	0.73	1.4 (0.58–3.38)	1.4 (0.57)	0.48 (−1.19)	1.4 (0.67)
Off label use	5	0.73	0.46 (0.19–1.11)	0.46 (3.16)	−1.11 (−2.78)	0.46 (0.22)
Abdominal pain	4	0.58	1.24 (0.46–3.31)	1.24 (0.18)	0.31 (−1.36)	1.24 (0.54)
Myalgia	4	0.58	1.97 (0.74–5.27)	1.96 (1.9)	0.97 (−0.7)	1.96 (0.86)
Eye pain	4	0.58	7.33 (2.74–19.6)	7.29 (21.72)	2.87 (1.2)	7.29 (3.2)
Decreased appetite	4	0.58	1.22 (0.46–3.27)	1.22 (0.16)	0.29 (−1.38)	1.22 (0.54)
Anxiety	4	0.58	1.11 (0.42–2.96)	1.11 (0.04)	0.15 (−1.52)	1.11 (0.49)
Abdominal pain upper	4	0.58	1.61 (0.6–4.31)	1.61 (0.93)	0.69 (−0.98)	1.61 (0.71)
Eye swelling	4	0.58	11.66 (4.36–31.18)	11.6 (38.67)	3.53 (1.86)	11.58 (5.08)
Dyspnoea	4	0.58	0.5 (0.19–1.35)	0.51 (1.93)	−0.98 (−2.65)	0.51 (0.22)
Chest discomfort	4	0.58	2.4 (0.9–6.42)	2.4 (3.26)	1.26 (−0.41)	2.4 (1.05)

In particular, we listed the signal strength of AEs at the PT level related to hearing and blood glucose change so as to provide a deeper insight into teprotumumab (Tables 6, 7). AEs related to hearing included tinnitus [ROR 39.92 (26.94–59.14)], deafness [ROR 106.77 (71.92–158.52)], ear discomfort [ROR 59.69 (30.82–115.59)], hypoacusis [ROR 17.73 (9.18–34.26)], ototoxicity [ROR 178.53 (56.46–564.54)], deafness unilateral [ROR 33.95 (8.44–136.59)], hyperacusis [ROR 31.06 (7.72–124.89)], deafness bilateral [ROR 52.86 (7.37–379.19)], ear disorder [ROR 17.41 (2.44–124.13)], autophony [ROR 2907.62 (263.33–32104.6)], and auditory disorder [ROR 43.72 (6.1–313.13)]. AEs related to blood glucose change included blood glucose increased [ROR 17.41 (10.89–27.82)], glycosylated haemoglobin increased [ROR 30.13 (13.46–67.45)], hyperglycaemia [ROR 11.97 (5.36–26.77)], decrease appetite [ROR 1.22 (0.46–3.27)], diabetes mellitus [ROR 1.53 (0.22–10.91)], polydipsia [ROR 27.3 (3.82–194.98)], blood glucose abnormal [ROR 6.68 (0.94–47.51)], and blood glucose fluctuation [ROR 11.31 (1.59–80.58)]. To sum up, the AEs analysis of real-world data based on the FAERS database could also provide a great reference for the instructions revision of teprotumumab.

**TABLE 6:** Signal strength of AEs of teprotumumab at the PTs level related to hearing in FAERS database.

PTs	n	ROR (95%Cl)	PRR (χ2)	IC (IC025)	EBGM (EBGM05)
Tinnitus	26	39.92 (26.94–59.14)	38.45 (942.96)	5.26 (3.59)	38.2 (27.49)
Deafness	26	106.77 (71.92–158.52)	102.78 (2575.8)	6.66 (4.99)	101.01 (72.57)
Ear discomfort	9	59.69 (30.82–115.59)	58.92 (507.39)	5.87 (4.19)	58.34 (33.56)
Hypoacusis	9	17.73 (9.18–34.26)	17.51 (139.82)	4.13 (2.46)	17.46 (10.06)
Ototoxicity	3	178.53 (56.46–564.54)	177.76 (511.64)	7.43 (5.74)	172.51 (65.84)
Deafness unilateral	2	33.95 (8.44–136.59)	33.86 (63.41)	5.07 (3.4)	33.67 (10.5)
Hyperacusis	2	31.06 (7.72–124.89)	30.97 (57.7)	4.95 (3.27)	30.81 (9.62)
Deafness bilateral	1	52.86 (7.37–379.19)	52.79 (50.35)	5.71 (4.02)	52.32 (10.06)
Ear disorder	1	17.41 (2.44–124.13)	17.39 (15.4)	4.12 (2.44)	17.34 (3.35)
Autophony	1	2907.62 (263.33–32104.6)	2903.39 (1934.26)	10.92 (8.71)	1935.93 (259.51)
Auditory disorder	1	43.72 (6.1–313.13)	43.66 (41.37)	5.44 (3.75)	43.34 (8.35)

**TABLE 7:** Signal strength of AEs of teprotumumab at the PTs level related to blood glucose in FAERS database.

PTs	n	ROR (95%Cl)	PRR (χ2)	IC (IC025)	EBGM (EBGM05)
Blood glucose increased	18	17.41 (10.89–27.82)	16.98 (270.26)	4.08 (2.41)	16.93 (11.44)
Glycosylated haemoglobin increased	6	30.13 (13.46–67.45)	29.88 (166.68)	4.89 (3.22)	29.73 (15.15)
Hyperglycaemia	6	11.97 (5.36–26.77)	11.88 (59.7)	3.57 (1.9)	11.86 (6.05)
Decreased appetite	4	1.22 (0.46–3.27)	1.22 (0.16)	0.29 (−1.38)	1.22 (0.54)
Diabetes mellitus	1	1.53 (0.22–10.91)	1.53 (0.19)	0.62 (−1.05)	1.53 (0.3)
Polydipsia	1	27.3 (3.82–194.98)	27.26 (25.18)	4.76 (3.08)	27.14 (5.24)
Blood glucose abnormal	1	6.68 (0.94–47.51)	6.67 (4.81)	2.74 (1.06)	6.66 (1.29)
Blood glucose fluctuation	1	11.31 (1.59–80.58)	11.3 (9.37)	3.5 (1.82)	11.28 (2.18)

### Time to onset analysis of teprotumumab-associated AEs

**FIGURE 5:**
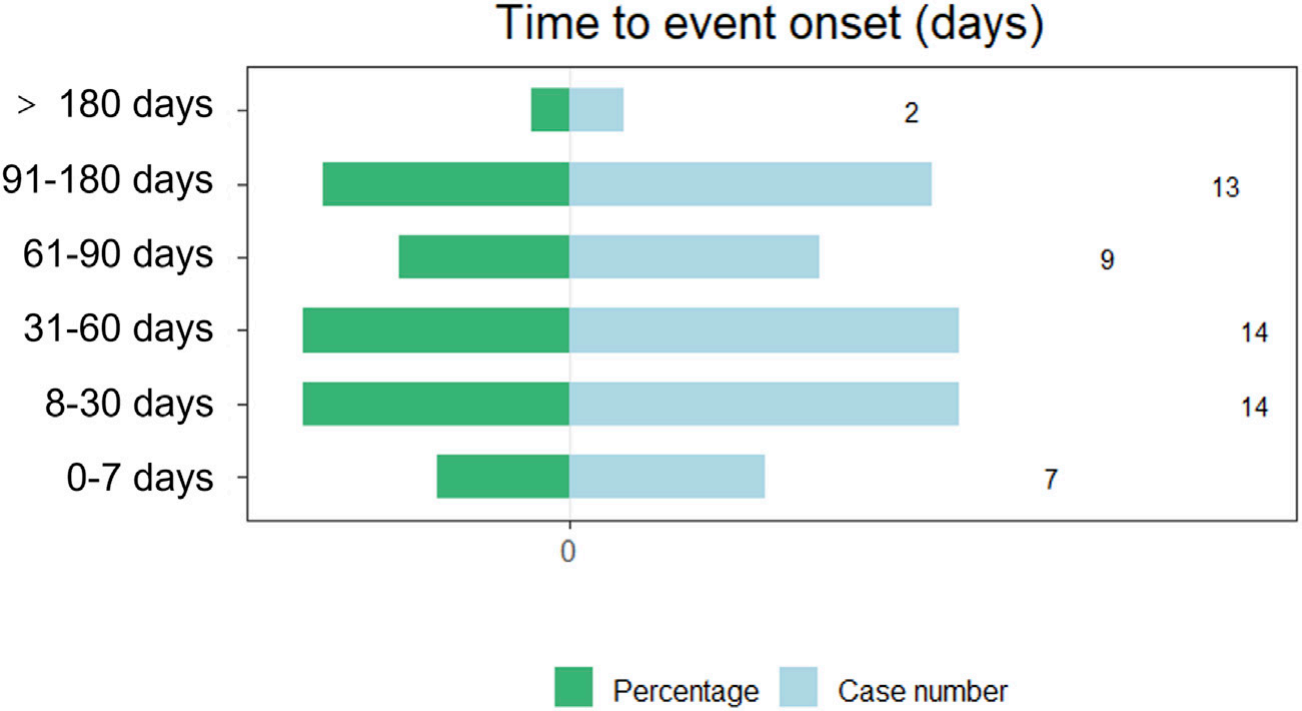
Time-to-onset of teprotumumab-related AEs.

The onset times of teprotumumab-related AEs were extracted and analyzed from the FAERS database. Overall, the median onset time of AEs associated with teprotumumab was 48 days (interquartile range [IQR] 19.0–92.0 days) after administering drugs. The times to onset of AEs for each administration of teprotumumab was also described (Figure 5). Approximately, one-third of the AEs occurred in 30 days (35.59%). Over half of the AEs occurred in 60 days (59.32%). Almost all the AEs occurred in 180 days (96.61%). Notably, data showed that AEs may still occur 180 days after teprotumumab treatment, which accounted for 3.39% of all AEs.

## Discussion

This study identified and characterized AEs that were significantly associated with teprotumumab from the FAERS database using ROR, PRR, BCPNN, and MGPS. The ROR method had the characteristics in the high sensitivity, and simplicity of calculation. However, it was easy to produce false positive signals when the number of reports was small. On the other hand, the Bayesian statistics, included BCPNN and MGPS, had characteristics in high specificity while the signal detection time was not as fast as frequentist statistics (Hauben and Bate, 2009). Each of these algorithms possesses unique strengths and limitations, and their selection depends on the requirement to strike a balance between sensitivity and specificity in detecting signals (Shu et al., 2022; Wang K. et al., 2024).

Teprotumumab, a monoclonal antibody that inhibits IGF-1R, is approved in the United States, Brazil and the Kingdom of Saudi Arabia under the brand name TEPEZZA^®^, where it is administered to patients through an intravenous infusion once every 3 weeks for a total of eight infusions (Smith and Janssen, 2019; Slentz et al., 2020). In March 2024, Amgen submitted a marketing authorization application to the Medicines and Healthcare products Regulatory Agency in Great Britain, a New Drug Submission to Health Canada and an application to the Therapeutic Goods Administration in Australia for teprotumumab. Teprotumumab is also under review by the Ministry of Health, Labour and Welfare in Japan. Teprotumumab has also recently been successfully introduced into China as a controlled medication and is accessible at the Boao Lecheng Weijian Rare Disease Clinical Medical Center. While it has not yet been approved by the National Medical Products Administration, patients can still receive treatment with the drug under specific medical institutions and conditions.

Teprotumumab has been shown to be effective in reducing proptosis and improving clinical activity score in patients with TED. However, teprotumumab also has some potential adverse effects that need to be considered. Based on the analysis, we put emphasis on those signals that were classified as strong signals in the AE reports of teprotumumab. In this case, it is important to conduct more research to evaluate the long-term safety and efficacy of teprotumumab in different populations and regions. Hopefully, our research will provide insight into drug applications in clinical practice, as well as drug import/export and development in different countries, aiming to improve the life quality of patients suffered from TED.

The data mining indicated that the primary SOCs for IGF-1R monoclonal antibody were gastrointestinal disorders, and ear and labyrinth disorders, and general disorders and administration site conditions, which was consistent with the primary adverse reactions listed in the instruction. However, the AEs analyzed in our study were far more complicated, which included nervous system disorders, and psychiatric disorders. The most common adverse effects reported in clinical trials were muscle spasms, nausea, alopecia, diarrhoea, fatigue, hyperglycaemia, hearing impairment, dysgeusia, headache, dry skin, and rash (Kahaly et al., 2021). The mechanism of these adverse effects is not fully understood, but may be related to the ubiquitous expression of IGF-1R and its role in various physiological processes.

Teprotumumab received approval based on clinical trials cited in the New England Journal of Medicine in 2017 and 2020. A combined analysis from these trials highlighted significant improvements observed 7 weeks after the final dose. During the 24-week double-masked period, most adverse events reported were mild to moderate. Among these, three events (4%) including diarrhea, infusion reaction, and Hashimoto’s encephalopathy (with associated confusion) were potentially related to teprotumumab and led to discontinuation from the study. Infusion reactions occurred in three patients, none of which were anaphylactic; two patients stopped treatment, while one continued with pre-medication and slower infusion rates. In the follow-up period beyond 24 weeks, two patients in the teprotumumab group experienced serious adverse events, including intercostal neuralgia and optic neuropathy, which were severe but not attributed to the study drug. Another serious, non-related event in the teprotumumab group was hypothyroidism. Additionally, two patients in this group had hyperglycemia-related adverse events, with one resolving without treatment and the other involving new-onset diabetes managed with metformin. Muscle spasms, considered mild and related to the study drug, resolved during follow-up. Other events such as hypoacusis and unilateral deafness showed varying degrees of improvement or resolution. Therefore, while teprotumumab offers therapeutic benefits, clinicians should carefully weigh these against potential risks, and closely monitor patients for adverse effects throughout treatment and follow-up (Kahaly et al., 2021).

In this study, we found many links between teprotumumab and systemic adverse events in pharmacovigilance analysis, which was consistent with the results of previous clinical studies. However, when dealing with a rare and possible adverse event issue, clinical cohorts and trials may not be able to provide a conclusive answer because of their strict selection criteria, small sample sizes, and short follow-up periods (Douglas et al., 2021; Kahaly et al., 2021; Winn and Kersten, 2021; Lin et al., 2023). The spontaneous reporting system could be a suitable source for new evidence. The possible mechanisms of these adverse events may be related to the inhibition of the IGF-1R by teprotumumab, which has a ubiquitous expression and a role in various physiological processes. For example, teprotumumab inhibits the IGF-1R, which is involved in glucose metabolism and insulin sensitivity. By blocking this receptor, teprotumumab may impair the ability of insulin to lower blood glucose levels, resulting in hyperglycemia. This effect is more pronounced in patients with diabetes or prediabetes, who may require adjustment of their antidiabetic medications. Muscle spasms may be due to the interference of teprotumumab with the normal function of IGF-1R in skeletal muscle, which is important for muscle growth, repair, and contraction. Inhibition of IGF-1R in skeletal muscle may cause muscle weakness, pain, and spasms. Hair loss may lie in the effect of teprotumumab on the expression of IGF-1R in hair follicles, which are responsible for hair growth and cycling. Hearing impairment and dysgeusia may result from the function of IGF-1R in the inner ear, which are involved in hearing. Menstrual changes may be due to the influence of teprotumumab on the production of IGF-1R in the ovaries, which are involved in ovarian function. In addition, teprotumumab may induce an immune response against the drug itself, as it is a foreign protein.

Our study has several strengths and limitations. One of the strengths is that we used four different disproportionality analysis algorithms to identify the significant signals of teprotumumab-related adverse events, which increased the robustness and reliability of our results. Another strength is that we used the FAERS database, which is a large and comprehensive database that collects spontaneous reports of adverse events from various sources, such as healthcare professionals, patients, manufacturers, and literature. However, this study has several limitations. Firstly, the FAERS database relies on voluntary reporting, leading to issues such as duplicate and incomplete reports. Many adverse event reports are submitted by patients, physicians, and company employees, resulting in potential double counting. Additionally, some reports lack crucial details, including comorbidities, relevant medical treatments, treatment dosages and durations. Secondly, the existence of a report does not establish causation, and rates of occurrence cannot be established from these reports. For any given report, it is uncertain whether the suspected drug caused the adverse event. Adverse events might be related to the underlying disease being treated, another drug being taken concurrently, or other factors. The reports reflect only the observations and opinions of the reporters. Therefore, the information provided cannot be used to estimate the incidence of the reported events or to ascribe causation to the drug. Thirdly, the information in these reports has not been verified. The submission of a report does not mean that the included information has been medically confirmed, nor does it imply that the reporter admits the drug caused or contributed to the event.

Our study has implications for clinical practice and future research. Our study provides valuable insight into the occurrence of adverse events following teprotumumab initiation, which can potentially support clinical monitoring and risk identification efforts. The use of teprotumumab should consider patient’s values and preferences in balancing the expected benefit with these potential risks. Patients receiving teprotumumab should be monitored for any signs of adverse events and receive appropriate treatment if needed. Future studies should investigate the mechanism, frequency, severity, and management of these adverse events, as well as the possible risk factors and predictors. Future studies should also explore the combination therapies with different agents that may maximize the benefits and minimize the risks of teprotumumab for the treatment of TED.

## Conclusion

In this study, we conducted a comprehensive assessment and analysis of post-marketing AEs associated with the IGF-1R monoclonal antibody, namely, teprotumumab. We used data mining methods on the FAERS database and applied four disproportionality analysis algorithms to identify the significant signals of teprotumumab-related adverse events, providing useful instructions for drug selection in clinical practice and valuable assistance in drug import/export and development.

## Data Availability

The original contributions presented in the study are included in the article/supplementary material, further inquiries can be directed to the corresponding author.

## Abbreviations

FDR, Food and drug administration; FAERS, Food and Drug Administration Adverse Event Reporting System; AE, Adverse Events; SMQs, Standardized MedDRA Queries; MedDRA, Medical Dictionary for Regulatory Activities; SOC, System Organ Class; PS, Primary suspect; TTO, time-to-onset; PTs, Preferred terms; ROR, Reporting odds ratio; PRR, Proportional reporting ratio; BCPNN, Bayesian confidence propagation neural network; MGPS, Multi-item gamma Poisson shrinker; CI, Confidence Interval; IC, Information Component; χ2, Chi-squared.
